# Magnetotransport and Magneto-Thermoelectric Properties of the Nodel-Line Semimetal SnTaS_2_

**DOI:** 10.3390/ma19030556

**Published:** 2026-01-30

**Authors:** Long Ma, Hao Tian, Xiaojian Wu, Dong Chen

**Affiliations:** 1 Shenzhen Skyworth Photovoltaic Technology Co., Ltd., Shenzhen 518083, China; malong@skyworth.com (L.M.); tianhao@skyworth.com (H.T.); wuxiaojian@skyworth.com (X.W.); 2 College of Physics, Qingdao University, Qingdao 266071, China

**Keywords:** nodal-line semimetal, magnetotransport, thermoelectric transport, quantum oscillations

## Abstract

Topological semimetals with nontrivial band structures host a variety of unconventional transport phenomena and have attracted significant attention in condensed matter physics. SnTaS_2_, a recently proposed topological nodal-line superconductor with a centrosymmetric layered structure, provides an ideal platform to explore the interplay between topology and electronic transport. Here, we report a comprehensive study of the normal-state magnetotransport and magneto-thermoelectric properties of SnTaS_2_ single crystals. We observed large magnetoresistance and nonlinear Hall resistivity at low temperatures, which can be well described by a two-band model, indicating the coexistence of electron and hole carriers. The Seebeck and Nernst coefficients were found to exhibit pronounced and nonmonotonic magnetic field dependences at low temperatures, consistent with multiband transport behavior. Moreover, clear quantum oscillations with a single frequency are detected in both electrical and thermoelectric measurements. Analysis of the oscillations reveals a small effective mass and a nontrivial Berry phase, suggesting that the corresponding Fermi surface arises from a topologically nontrivial band. These findings shed light on the normal-state electronic structure of SnTaS_2_ and highlight the important role of topological bands in shaping its transport properties.

## 1. Introduction

Due to the nontrivial band structures, topological semimetals exhibit a wealth of novel physical phenomena, attracting extensive attention in the condensed matter physics and materials science communities [1,2]. According to their band structure near the Fermi level (*E*_F_), topological semimetals can be classified into Dirac semimetals, Weyl semimetals, nodal-line semimetals, et al., such as Na_3_Bi, TaAs, and ZrSiS [3,4,5]. A common feature of these band structures is the presence of linear band crossings near the *E*_F_, which leads to a variety of unconventional transport properties, including extremely large magnetoresistance (MR), negative MR induced by the chiral anomaly, giant anomalous Hall effect, and giant anomalous Nernst effect [6,7,8,9]. Owing to these exotic properties, topological semimetals hold great potential for applications in spintronics and thermoelectric energy conversion.

Recently, a novel topological nodal-line superconductor SnTaS_2_ has attracted considerable attention [10,11,12,13,14,15,16,17,18,19]. SnTaS_2_ is a layered material with a centrosymmetric crystal structure. First-principles calculations have revealed the presence of a nodal ring near the Fermi level in its band structure, along with the associated drumhead-like topological surface states [10]. These band-structure features were subsequently confirmed by angle-resolved photoemission spectroscopy (ARPES) and electrical transport measurements [12,14]. The coexistence of superconductivity and topological surface states is expected to provide a promising platform for realizing Majorana zero modes, and thus holds potential for topological quantum computation [20]. Although SnTaS_2_ has been extensively investigated, most previous studies have focused on its superconducting state, while the physical properties of the normal state remain relatively unexplored.

In this work, we present a comprehensive study of the normal-state magneto-transport and magneto-thermoelectric properties of bulk single-crystalline SnTaS_2_. By combining electrical transport, Hall effect, Seebeck and Nernst measurements with quantum oscillation analysis, we provide a unified view of charge and entropy transport in this nodal-line system. Pronounced quantum oscillations are observed in both electrical and thermoelectric channels, allowing us to extract the cyclotron effective mass and a nontrivial Berry phase in the normal state. Furthermore, the apparent sign mismatch between the Hall and Seebeck coefficients is discussed in the context of multiband transport and Fermi-surface geometry. Our results demonstrate that magneto-thermoelectric transport offers a sensitive probe of topological electronic structures beyond conventional electrical measurements, and provide new insight into the normal-state properties of topological nodal-line materials.

## 2. Materials and Methods

Single crystals of SnTaS_2_ were grown by the chemical vapor transport method. Sn powder, Ta powder, and S powder with atomic ratio of 0.33:1:2 was mixed and thoroughly ground, pressed into pellets, and sealed in an evacuated quartz tube. The tube was heated at 850 °C for 2 days to synthesize the Sn_0.33_TaS_2_ precursor. The obtained Sn_0.33_TaS_2_ powder was mixed with additional Sn powder to achieve a molar ratio of Sn:Ta:S = 1.2:1:2, and sealed in an evacuated quartz tube together with iodine at a concentration of 3 mg/mL. The tube was subsequently placed in a two-zone furnace, with the source zone maintained at 1000 °C and the growth zone at 970 °C for 2 weeks. Plate-like single crystals were obtained at the growth zone with typical dimensions of about 3 × 3 × 0.01 mm^3^. The crystal structure of the grown single crystals was characterized by X-ray diffraction (XRD) on a Rigaku Smartlab X-ray diffractometer with Cu Kα radiation at room temperature (Rigaku, Tokyo, Japan). The elemental composition was determined by energy-dispersive X-ray (EDX) spectroscopy performed on clean areas of the crystals by a Bruker XFlash 6-100 detector (Bruker, Billerica, MA, USA) attached to a Zeiss scanning electron microscope (Gemini 360, ZEISS, Oberkochen, Germany). The electrical transport properties were measured using a Quantum Design physical property measurement system (PPMS-9, Quantum Design, San Diego, CA, USA). The thermoelectric measurements were performed with a self-built one-heater-two-thermometers setup based on the PPMS system.

## 3. Results and Discussion

**Figure 1:**
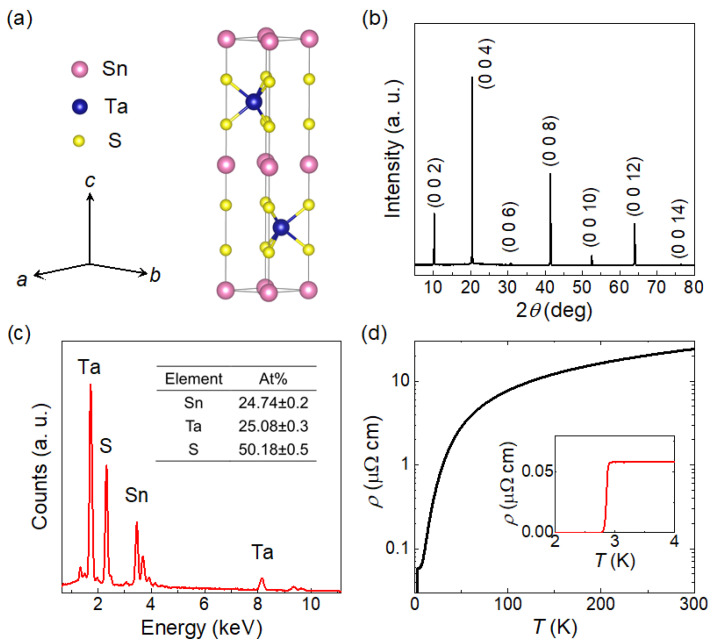
(**a**) Crystal structure of SnTaS_2_. (**b**) XRD pattern collected from the largest surface of a SnTaS_2_ single crystal, where all peaks can be indexed to the (00*l*) reflections. (**c**) EDX spectrum of a representative single crystal, showing an approximate atomic ratio of Sn:Ta:S = 1:1:2. (**d**) Temperature dependence of the resistivity. The inset shows an enlarged view of the low-temperature region.

Figure 1a shows the crystal structure of SnTaS_2_, which is a centrosymmetric layered structure with space group *P*6_3_/*mmc*. This structure consists of alternately stacked TaS_2_ layers and Sn layers, with lattice parameters *a* = *b* = 3.309 Å, and *c* = 17.450 Å. Figure 1b displays the XRD pattern taken from the largest surface of a single crystal. All diffraction peaks can be well indexed to the (00l) reflections of SnTaS_2_, indicating that the largest surface of the crystals corresponds to the *ab* plane. Figure 1c presents the EDX spectrum of the crystal, showing an approximate elemental ratio of Sn:Ta:S = 1:1:2. Since SnTaS_2_ is a superconductor, we further measured the temperature-dependent resistivity, as shown in Figure 1d. The resistivity exhibits a monotonic metallic behavior with a residual resistivity ratio RRR = *ρ* (300 K)/*ρ* (4 K) = 415, indicating the high quality of the crystals. A superconducting transition is observed at 3 K, as highlighted in the inset. All these characterizations are consistent with previous reports [10,11,16].

**Figure 2:**
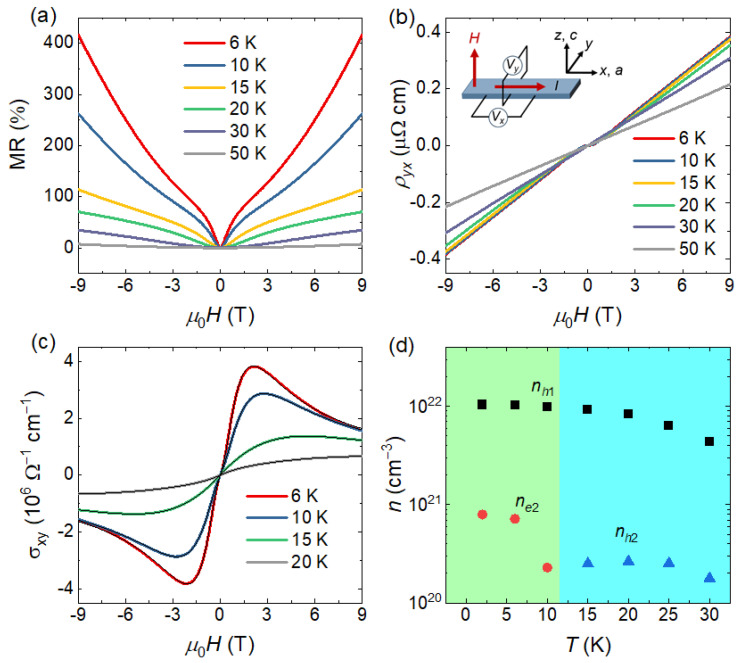
(**a**) Magnetic field dependence of the MR measured at different temperatures. (**b**) Magnetic field dependence of the Hall resistivity $\rho_{yx}$ at different temperatures. The inset shows the measurement configuration with the magnetic field applied along the *c* axis and the electric current along the *a* axis. (**c**) Hall conductivity $\sigma_{xy}$ as a function of magnetic field at selected temperatures, together with the black curves of two-band fitting. (**d**) Temperature dependence of the carrier concentrations extracted from the two-band fitting, showing one dominant hole-type carrier (*n_h_*_1_) over the whole temperature range accompanied by an electron-type carrier (*n_e_*_2_) at low temperatures and a hole-type carrier (*n_h_*_2_) at high temperatures.

Figure 2a,b show the MR and Hall resistivity measured at different temperatures. The measurement geometry is illustrated by the inset of Figure 2b, with the magnetic field applied along the *c* axis, the current along the *a* axis, and the voltage contacts for the longitudinal and Hall resistivities arranged parallel and perpendicular to the *a* axis, respectively. The MR is defined as $MR=[\rho_{xx}(B)-\rho_{xx}(0)]/\rho_{xx}(0)\times 100\%$. At low temperatures, the MR exhibits a pronounced dip at low magnetic fields, which may originate from the weak antilocalization effect [16]. The MR reaches as high as 400% under a magnetic field of 9 T at *T* = 6 K, which is significantly larger than the typical few-percent MR observed in conventional metals. Such a large MR effect has been widely reported in many semimetals and is commonly attributed to the compensation between coexisting electron-type and hole-type carriers [21]. This scenario can be confirmed by the field-dependent Hall resistivity $\rho_{yx}(B)$ at different temperatures, as shown in Figure 2b. It is clearly observed that below 30 K, the Hall resistivity exhibits a nonlinear field dependence near zero field, which is characteristic of multicarrier transport. In particular, the Hall resistivity below 15 K shows a negative slope near zero magnetic field but a positive slope at higher fields, providing clear evidence for the coexistence of electron and hole carriers.

To further extract the carrier information, we first calculated the Hall conductivity at different temperatures using $\sigma_{xy}(B)=\frac{\rho_{yx}(B)}{\rho_{yx}^{2}(B)+\rho_{xx}^{2}(B)}$. The resulting Hall conductivity was then fitted using a semiclassical two-band model [22]

$$\sigma_{xy}=\left[\frac{n_{1}\mu_{1}^{2}}{1+(\mu_{1}B{)}^{2}}+\frac{n_{2}\mu_{2}^{2}}{1+(\mu_{2}B{)}^{2}}\right]eB,$$

where $n_{1}$($n_{2}$) and $\mu_{1}$($\mu_{2}$) are the carrier density and mobility of the first (second) type of carriers, respectively. The positive (negative) sign of the resulting $n_{1}$ or $n_{2}$ corresponding to hole-type (electron-type) carriers. Figure 2c shows the Hall conductivity at selected temperatures together with the fitting lines, demonstrating that the two-band model provides an excellent description of the experimental data. Figure 2d presents the temperature dependence of the carrier concentrations extracted from the two-band fitting. We can observe that the system always hosts a hole-type carrier with a relatively large carrier concentration *n_h_*_1_, along with an electron-type carrier with a much smaller concentration *n_e_*_2_ at and below 10 K, and a hole-like carrier with concentration *n_h_*_2_ at higher temperatures. In general, the carrier concentration in a semimetal is expected to increase with increasing temperature due to thermal excitation. However, our results show that the carrier concentration decreases with increasing temperature below 30 K. The reason for the changed carrier type and the anomalous reduction in carrier concentrations need further investigation. One possible explanation for these phenomena may be the temperature-induced Lifshitz transition [23,24,25,26]. It is worth noting that, after subtracting the background, quantum oscillations are clearly observed in both the field-dependent $\rho_{xx}$ and $\rho_{yx}$ at low temperatures. The quantum oscillations will be discussed in detail below.

**Figure 3:**
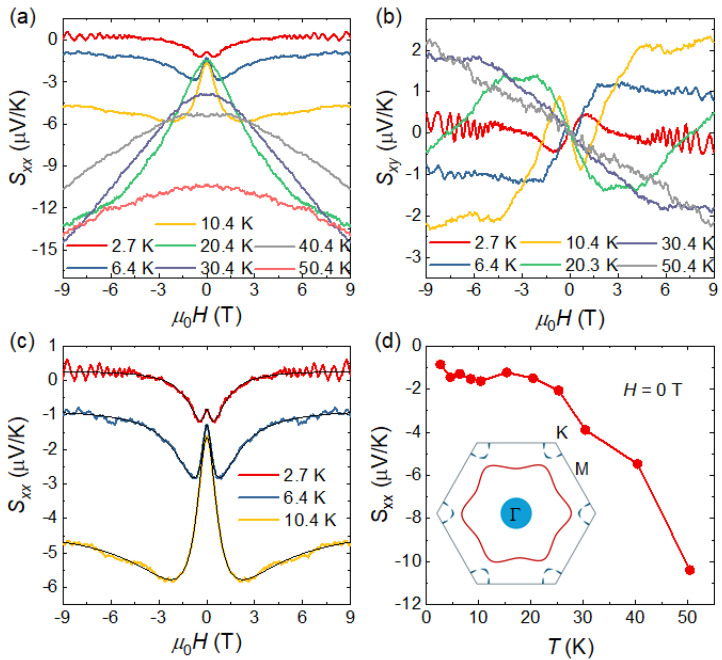
(**a**,**b**) Magnetic field dependence of the Seebeck coefficient $S_{xx}$ and the Nernst coefficient $S_{xy}$ at different temperatures, respectively. (**c**) Representative fitting (black lines) of the field-dependent Seebeck coefficient using a multiband model at low temperatures, demonstrating good agreement with the experimental data. (**d**) Temperature dependence of the zero-field Seebeck coefficient. The inset illustrates the Fermi surface of SnTaS_2_ obtained from first-principles calculations.

Figure 3a,b show the magnetic-field dependence of the Seebeck coefficient $S_{xx}=\frac{E_{x}}{\nabla_{x}T}$, and the Nernst coefficient $S_{xy}=\frac{{-E}_{y}}{\nabla_{x}T}$ of SnTaS_2_ single crystals at different temperatures, respectively. The temperature gradient is applied along the *a* axis, and the Seebeck and Nernst voltage contacts are applied parallel and perpendicular to the *a* axis, similar to that for the magnetotransprot measurements (see the inset of Figure 2a). The Seebeck and Nernst effects correspond to thermoelectric responses in which an electric potential is generated parallel and perpendicular to the applied temperature gradient, respectively. Both of the *S_xx_* and *S_xy_* show a nonmonotonic field dependence at low temperatures, indicating the multiband nature of the system, especially for the *S_xx_*. The sign of *S_xx_* reflects the dominant carrier type, that is, negative values (positive) indicate electron-type (hole-type) carriers. When the temperature is below or equal to 10 K, the *S_xx_* exhibits a mustache-shaped field dependence, which cannot be explained by a simple single-band model [27]. Considering the two-band nature of this system revealed previously, we fitted the data using the following semiclassical expression [28]:

$$S_{xx}(B)=S_{1}\frac{1}{1+{\left(\mu_{1}B\right)}^{2}}+S_{2}\frac{1}{1+{\left(\mu_{2}B\right)}^{2}}+S_{\infty }\frac{{\left(\mu^{\prime }B\right)}^{2}}{1+{\left(\mu^{\prime }B\right)}^{2}},$$

where *S*_1_ (*S*_2_) and *μ*_1_ (*μ*_2_) are the zero-field Seebeck coefficients and mobility of the first (second) carrier, respectively, and $S_{\infty }$ is the limiting value when *B* tends to infinity. This expression reproduces the experimental curves well, as shown in Figure 3c.

Table 1 summrizes the fitting results for the Seebeck coefficient, together with the parameters obtained from the two-band fitting of the Hall conductivity at two representative temperatures of 6 and 10 K. Although both the Hall and Seebeck measurements indicate the coexistence of electron and hole type carriers, the dominant carriers inferred from the two responses are different. Specifically, the Hall conductivity is dominated by hole-type carriers, whereas the Seebeck coefficient is dominated by electron-type carriers. Moreover, the carrier mobilities extracted from the two measurements also differ. This discrepancy is further reflected by the zero-field Seebeck coefficient, as shown in Figure 3d. It remains negative over the entire measured temperature range, with its absolute value increasing slowly with temperature below 20 K and rising rapidly above 20 K. The negative Seebeck coefficient indicates that electron-type carriers are dominant, which is inconsistent with the Hall measurement results suggesting hole-type carriers as the majority. This discrepancy may arise from the presence of a two-dimensional (2D) Fermi surface with a special geometry [12,14,29,30]. The inset of Figure 3d illustrates the Fermi surface of SnTaS_2_, which mainly consists of a 3D hole pocket centered at the Γ point, a large 2D hexagonal warped electron Fermi surface, and several small pockets around the K points with both electron and hole characters. Among these features, the hexagonally warped electron Fermi surface has the largest volume and is therefore expected to dominate the transport behaviors, which is consistent with the negative Seebeck coefficient observed over the entire measured temperature range. However, the sign of the Hall effect is also affected by the geometry of the Fermi surface. In particular, in materials whose Fermi surfaces contain regions with both positive and negative curvature, the Hall coefficient can exhibit a sign opposite to that inferred from the dominant carrier type. The two-band models employed here are semiclassical and phenomenological frameworks, not a fully microscopic description [31]. A full quantitative treatment of this effect lies beyond the scope of the present work and will require further investigation.

**Figure 4:**
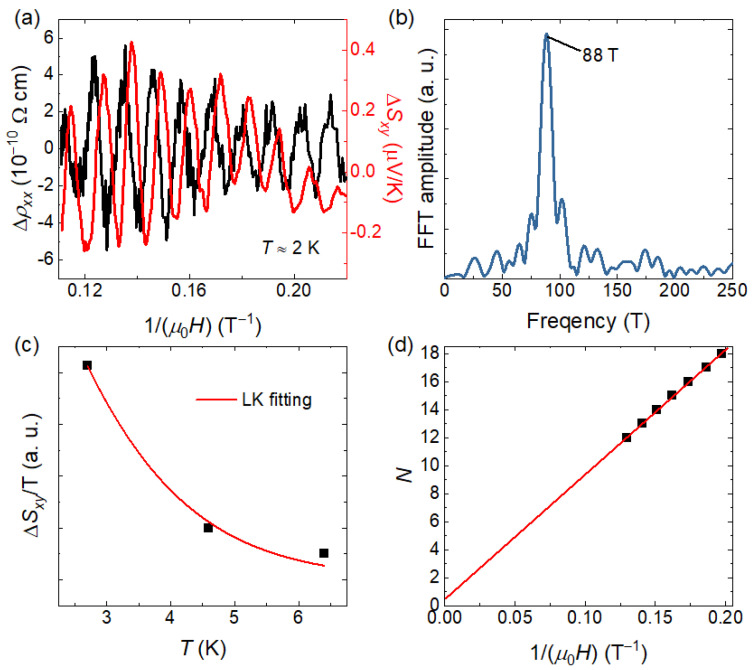
(**a**) Quantum oscillation parts of $\rho_{xx}$ and *S_xy_* as a function of inverse magnetic field. (**b**) FFT spectrum of the quantum oscillations of $\rho_{xx}$, revealing a single dominant frequency. (**c**) Temperature dependence of the amplitude of the thermoelectric quantum oscillations, fitted using the LK formula to extract the cyclotron effective mass. (**d**) Landau index plot constructed by the minima ${∆\sigma }_{xx}$. The linear extrapolation yields an intercept close to $\frac{1}{2}$, indicating a nontrivial Berry phase associated with a topologically nontrivial band.

It can be observed that both the Seebeck and Nernst coefficients exhibit quantum oscillations at low temperatures. In addition, quantum oscillations can also be resolved in the electrical transport data after subtracting the background. All the background is obtained by polynomial fitting in the field range of 4–9 T. Figure 4a shows the quantum oscillations of the resistivity and the Nernst coefficient as a function of inverse magnetic field. Both signals exhibit a single dominant frequency, differing only by a phase shift. Quantum oscillations can provide further information about the electronic band structure. According to the Onsager relation, the frequency of quantum oscillations is directly related to the extremal cross-sectional area of the Fermi surface, $F=(\Phi_{0}/2\pi^{2})A_{F}$, where F is the oscillation frequency, *A_F_* is the Fermi-surface cross-sectional area, and $\Phi_{0}$ is the magnetic flux quantum [32]. We therefore performed fast Fourier transform (FFT) of the quantum oscillations in the resistivity, as shown in Figure 4b. The FFT was carried out with a rectangle window and a sampling interval of 2 × 10^−4^. The FFT spectrum contains only a single dominant frequency at 88 ± 0.3 T. According to the Onsager relation, this frequency corresponds to a Fermi surface with cross-sectional area of *A_F_* = (8.4 ± 0.03) × 10^−3^ Å^−2^, occupying 0.2% of the cross-sectional area of the first Brillouin zone. Due to the larger oscillation amplitude in the thermoelectric transport, we further extracted the cyclotron effective mass of this Fermi surface using the Lifshitz–Kosevich (LK) theory. For quantum oscillations in thermoelectric coefficients, the LK formula can be written as [33]:

$$\frac{A}{T}\propto \frac{2\pi^{2}k_{B}Tm^{*}/eB\hbar }{\sinh\left(2\pi^{2}k_{B}Tm^{*}/eB\hbar \right)},$$

where *A* is the oscillation amplitude of ${∆S}_{xx}$ or ${∆S}_{xy}$, and $m^{*}$ is the cyclotron effective mass. *B* is determined by the range of oscillation: 1/*B* = (1/*B*_1_ + 1/*B*_1_)/2, where 1/*B*_1_ = 0.11 T^−1^ and 1/*B*_2_ = 0.25 T^−1^ are the lower and upper bounds of 1/*B*. The fitting result is shown in Figure 4c, yielding an effective mass of $m^{*}=0.50\pm 0.05\;m_{e}$.

Finally, we analyze the topology of the band corresponding to the main frequency. In Shubnikov–de Haas (SdH) oscillations, the conductivity oscillates as [34]:

$$\Delta \sigma_{xx}∼cos\left[2\pi \left(\frac{F}{B}-\frac{1}{2}+\beta +\delta \right)\right],$$

where β is the Berry phase divided by 2π, and δ is a phase shift that takes values of 0 for two-dimensional systems and $\pm \frac{1}{8}$ for three-dimensional systems. To determine β, we calculated the longitudinal conductivity $\sigma_{xx}(B)=\frac{\rho_{xx}(B)}{\rho_{yx}^{2}(B)+\rho_{xx}^{2}(B)}$, subtracted the background to obtain $\Delta \sigma_{xx}$, and constructed the Landau index plot by assigning integer indices to the minima of $\Delta \sigma_{xx}$, as shown in Figure 4d. With this convention, an intercept on the index axis of $\frac{1}{2}$ (0) will correspond to a nontrivial (trivial) Berry phase of π (0). By a linear fitting, the extrapolated intercept on the index axis is 0.5 ± 0.04. In the current stage, we have not enough evidence for the dimension of the Fermi surface corresponding to this oscillation. The phase shift δ could be either 0 or $\pm \frac{1}{8}$. However, regardless of the dimensionality of this Fermi surface, it can certainly yield a nontrivial Berry phase close to π within the uncertainty of the intercept, and suggest the topologically nontrivial nature of the associated band structure. We also tried another indexing convention by assigning half-integer indices to the maximum of $\Delta \sigma_{xx}$, which also leads to a consistent result.

This dominant oscillation frequency at 88 T has also been reported in previous de Haas–van Alphen (dHvA) measurements on bulk crystals and SdH measurements on thin films of SnTaS_2_ [11,13]. For this oscillation frequency, previous studies did not find a corresponding Fermi surface within the bulk band structure, whereas a topological surface state with a similar cross-sectional area was found. Since our experiments are performed on bulk single crystals, the pronounced quantum oscillations observed in the thermoelectric coefficients tend to suggest a bulk origin of the oscillations. However, the contribution from surface states cannot be completely excluded in the current stage. We hope that our results provide additional useful information for further identifying the origin of this oscillation. Further investigations on this issue, such as angle-dependent quantum oscillation measurements, are required in the future.

## 4. Conclusions

In conclusion, we have systematically investigated the normal-state magnetotransport and magneto-thermoelectric transport properties of the topological nodal-line superconductor SnTaS_2_. A giant magnetoresistance and nonlinear Hall resistivity are observed, which can be well understood in terms of the coexistence of electron-type and hole-type carriers within a multiband framework. At low temperatures, both the Seebeck and Nernst coefficients exhibit nonmonotonic magnetic-field dependence, further supporting the multiband nature of the electronic transport. The Hall and Seebeck measurements yield opposite dominant carrier types, which may originate from the unusual Fermi surface geometry. Moreover, pronounced quantum oscillations with a single dominant frequency are observed in both electrical and thermoelectric transport measurements. Analysis based on the Lifshitz–Kosevich theory yields a moderate cyclotron effective mass, and the Landau index plot reveals a nontrivial Berry phase, indicating a topological nontrivial band. These results provide important insights into the normal-state electronic structure of SnTaS_2_ and establish a solid foundation for understanding the interplay between topology and superconductivity in this material.

## Figures and Tables

**Table 1:** Comparison of the parameters obtained from two-band fitting of the Hall conductivity and Seebeck coefficient.

*T* (K)	*n*_*h*1_ (cm^−3^)	*μ*_*h*1_ (cm^2^/Vs)	*n*_*e*2_ (cm^−3^)	*μ*_*e*2_ (cm^2^/Vs)	*S*_*h*1_ (μV/K)	*μ*_*h*1_ (cm^2^/Vs)	*S*_*e*2_ (μV/K)	*μ*_*e*2_ (cm^2^/Vs)	*S*_∞_ (μV/K)
6	(1.04 ± 0.003) × 10^22^	5252 ± 17	(7.17 ± 0.4) × 10^20^	15,266 ± 344	1.71 ± 0.12	24,684 ± 56	−3.0 ± 0.11	5534 ± 128	−0.90 ± 0.08
10	(9.95 ± 0.004) × 10^21^	3770 ± 4	(2.29 ± 0.05) × 10^20^	15,623 ± 219	1.02 ± 0.07	13,158 ± 37	−2.68 ± 0.06	2511 ± 62	−4.26 ± 0.46

## Data Availability

The original contributions presented in this study are included in the article. Further inquiries can be directed to the corresponding author.
